# Nutrition and inflammation serum biomarkers are associated with 12-week mortality among malnourished adults initiating antiretroviral therapy in Zambia

**DOI:** 10.1186/1758-2652-14-19

**Published:** 2011-04-10

**Authors:** John R Koethe, Meridith Blevins, Christopher Nyirenda, Edmond K Kabagambe, Bryan E Shepherd, C William Wester, Isaac Zulu, Janelle M Chiasera, Lloyd B Mulenga, Albert Mwango, Douglas C Heimburger

**Affiliations:** 1 Centre for Infectious Diseases Research in Zambia, Plot 1275 Lubuto Road, Lusaka, Zambia; 2 Vanderbilt Institute for Global Health, 2525 West End Ave, Nashville, TN 37203, USA; 3 Department of Medicine, Vanderbilt University School of Medicine, MCN D-3100, 1161 21st Ave, Nashville, TN 37232, USA; 4 Department of Biostatistics, Vanderbilt University School of Medicine, MCN S-2323, 1161 21st Ave, Nashville, TN 37232, USA; 5 Department of Internal Medicine, University Teaching Hospital, Private Bag RWIX, Lusaka, Zambia; 6 Harvard School of Public Health, Division of Immunology and Infectious Diseases, 677 Huntington Avenue, Boston, MA 02115, USA; 7 Zambian Ministry of Health, Ndeke House, PO Box 30205, Lusaka, Zambia; 8 Department of Epidemiology, University of Alabama at Birmingham, Room 220, 1665 University Blvd, Birmingham, AL 35294, USA; 9 Department of Clinical Laboratory Sciences, University of Alabama at Birmingham, SHPB 433, 1530 3rd Ave South, Birmingham, AL 35294, USA; 10 Department of Nutrition Sciences, University of Alabama at Birmingham, LRC 354B, 1714 9th Avenue South, Birmingham, AL 35294-3412, USA

## Abstract

**Background:**

A low body mass index (BMI) at antiretroviral therapy (ART) initiation is a strong predictor of mortality among HIV-infected adults in resource-constrained settings. The relationship between nutrition and inflammation-related serum biomarkers and early treatment outcomes (e.g., less than 90 days) in this population is not well described.

**Methods:**

An observational cohort of 142 HIV-infected adults in Lusaka, Zambia, with BMI under 16 kg/m^2 ^or CD4^+ ^lymphocyte counts of less than 50 cells/mm^3^, or both, was followed prospectively during the first 12 weeks of ART. Baseline and serial post-treatment phosphate, albumin, ferritin and highly sensitive C-reactive protein (hsCRP) serum levels were measured. The primary outcome was mortality.

**Results:**

Lower baseline phosphate and albumin serum levels, and higher ferritin and hsCRP, were significantly associated with mortality prior to 12 weeks (p < 0.05 for all comparisons), independent of known risk factors for early ART-associated mortality in sub-Saharan Africa. The time-dependent interval change in albumin was associated with mortality after adjusting for the baseline value (AHR 0.62 [0.43, 0.89] per 5 g/L increase), but changes in the other biomarkers were not.

**Conclusions:**

The predictive value of serum biomarkers for early mortality in a cohort of adults with malnutrition and advanced HIV in a resource-constrained setting was primarily driven by pre-treatment values, rather than post-ART changes. Interventions to promote earlier HIV diagnosis and treatment, address nutritional deficiencies, and identify the etiologies of increased systemic inflammation may improve ART outcomes in this vulnerable population.

## Background

The combination of untreated HIV infection and a poor nutritional state interact in a complex cycle that hastens both immunosuppression and weight loss, and a low body mass index (BMI) (less than 18.5 kg/m^2^) is a powerful predictor of early mortality following antiretroviral therapy (ART) initiation in several reports from resource-constrained settings [1-4]. A reduced serum albumin level, potentially due to inflammatory cytokine-induced inhibition of protein synthesis, inadequate nutritional intake, or other causes, is also associated with increased mortality in HIV-infected adults [5-8]. Similarly, heightened systemic inflammation, including elevated serum levels of C-reactive protein (CRP) and ferritin (an acute phase reactant), is associated with accelerated loss of lean body mass and a more rapid progression to AIDS and death [9-12].

We previously demonstrated that serum phosphate levels at ART initiation are independently and negatively associated with early mortality in an observational cohort of severely malnourished (BMI below 16 kg/m^2^) and/or immunosuppressed (CD4^+ ^lymphocyte count below 50 cells/mm^3^) adults initiating treatment in Lusaka, Zambia [13]. A central hypothesis was that a variant of the refeeding syndrome, or an early, precipitous drop in serum phosphate in response to increased metabolic activity and/or carbohydrate intake among malnourished individuals starting ART contributed to the high rates of early mortality among low BMI adults in sub-Saharan Africa [14,15]. However, we found no association between one-week post-ART phosphate levels and subsequent mortality, suggesting that acute electrolyte derangements were not a proximate cause of death in most instances.

Prior studies have demonstrated associations between long-term (i.e., more than six months) changes in inflammatory biomarkers or albumin, and subsequent health outcomes in persons living with HIV [16,17], but the relationship between these biomarkers and mortality in the immediate post-ART period is not well studied, especially in resource-constrained settings. In this analysis, we describe relationships between pre-treatment levels and post-ART changes in serum phosphate, albumin, ferritin and CRP and the risk of mortality in the first 12 weeks of ART in a cohort of adults in sub-Saharan Africa with advanced malnutrition and immunosuppression.

## Methods

We enrolled 142 HIV-infected adults with a BMI of less than 16 kg/m^2 ^or a CD4^+ ^lymphocyte count below 50 cells/mm^3 ^initiating ART at a public sector clinic in Lusaka, Zambia, in an observational, prospective cohort study to assess metabolic predictors of all-cause mortality in the first 12 weeks of treatment. The study setting, eligibility criteria, design and procedures have been previously described [13]. Briefly, individuals were eligible for enrolment if they: qualified for ART according to Zambian national guidelines in place at the time (i.e., WHO stage 4 disease, a CD4^+ ^lymphocyte count below 200 cells/mm^3^, or WHO stage 3 disease and a CD4^+ ^lymphocyte count below 350 cells/mm^3^); were intending to start therapy the same day; met the BMI and/or CD4^+ ^threshold criteria; and agreed to adhere to the study visit schedule and laboratory testing requirements.

At the enrolment visit and the subsequent study visits at one, two, four, eight and 12 weeks post-ART initiation, participants were evaluated by a research nurse and a clinical officer and/or a supervising physician. The first-line ART regimen was selected from the national programme formulary: two nucleoside reverse transcriptase inhibitors in combination with one non-nucleoside reverse transcriptase inhibitor.

At the initial visit, a detailed health history, review of systems, physical examination and laboratory testing (including a basic metabolic panel, serum phosphate, albumin, ferritin and highly-sensitive C-reactive protein) were performed. Phosphate and albumin were measured at each subsequent study visit, while ferritin measurements were repeated at one, two, four and 12 weeks, and highly sensitive C-reactive protein (hsCRP) measurements were repeated at one, four and 12 weeks post-ART initiation. Thirteen participants received phosphate supplementation according to predetermined algorithm based on serum level [13].

Participant deaths were reported to the study staff by the health clinic. If a participant missed a study visit and could not be contacted by phone, community outreach teams attempted to locate the individual or a close relative to determine the vital status [18]. If the participant could not be located and vital status was unknown, he or she was deemed lost to follow up.

Routine and study-specific chemistry assays were measured using a Roche COBAS Integra 400+ (Roche Diagnostics, Basel, Switzerland) or a Pointe 180 Chemistry Analyzer (Pointe Scientific, Canton, MI, USA). CD4^+ ^lymphocyte counts were performed using a Beckman Coulter Epics XL-MCL flow cytometer (Beckman Coulter, Inc., Fullerton, CA, USA), and hemogram using a Horiba ABX Pentra 80 (Horiba ABX Diagnostics Inc., Montpellier, France).

Cox proportional hazards models were used to assess the relationship between the baseline nutrition or inflammation serum biomarker values and time to death. The biomarkers were modelled as continuous variables and expanded using restricted cubic splines to avoid linearity assumptions. To reduce the number of variables in the Cox models, we used a principal component analysis with age, hemoglobin, CD4^+ ^lymphocyte count and BMI to extract the first principal factor to represent these variables at baseline. All Cox models were adjusted for sex and this principal component.

If the vital status at 90 days was unknown (i.e., lost to follow up), the participant was censored at the last study visit date. A second multivariable regression used similar techniques to model the relationship between the baseline biomarkers and a composite endpoint of time to death or loss to follow up. We accounted for missing baseline status values (29 hemoglobin, two ages, and one BMI value) and serum biomarkers (eight phosphate, 22 albumin, and 58 hsCRP and ferritin values) using multiple imputation techniques [19]. The proportion of imputed values was within the commonly accepted range [19].

To visualize longitudinal data, we plotted the serum indicators of interest for the alive, deceased and deceased/lost cohorts against the number of days on ART; each participant required a minimum of two recorded values for inclusion (participants who had only one recorded value, generally because they had died, were excluded). For each variable, we sketched a locally weighted scatterplot smoothing (LOWESS) curve by fitting a polynomial surface using local (weighted) least squares regression [20].

Cox models with baseline and time-dependent covariates were used to assess the relationship between serial biomarker values and time to death, or the composite endpoint of time to death or loss to follow up. Each biomarker was modelled separately, and the model was adjusted for the biomarker baseline serum value, in addition to sex and the principal component (as already described). If the participant had no recorded serum level within a given time interval (i.e., week one visit, week four visit, etc.), he or she was dropped from the risk set for that interval. R-software 2.11.0 (http://www.r-project.org) was used for data analyses.

The study protocol and informed consent documents were approved by the University of Zambia Research Ethics Committee (Lusaka, Zambia), and the Institutional Review Boards at the University of Alabama at Birmingham (Birmingham, Alabama, USA) and Vanderbilt University (Nashville, Tennessee, USA).

## Results

We enrolled 142 participants between 6 November 2006 and 12 November 2007; 59 (42%) participants had BMI below 16.0 kg/m^2 ^and 110 (77%) had CD4^+ ^lymphocyte counts below 50 cells/mm^3 ^(27, or 19%, met both eligibility criteria). Twenty-five participants died over the 12-week follow-up period (mortality rate: 87.4 per 100 person-years of follow up); 10 (40%) participants died within four weeks of starting ART, although none died in the first week. The median time to death was 34 days (interquartile range [IQR]: 20, 54). Thirty-three participants (23%) were lost to follow up; the median follow-up time for those lost was 58 days (IQR: 45, 71). Table 1 describes participant characteristics, baseline serum biomarker levels, and the distribution of first-line ART regimens.

**Table 1:** Baseline participant characteristics and serum biomarker values

Participant demographics and clinical characteristics		
**N**	142	

**Female**, n (%)	87 (61%)	

**Age**, median years (IQR)	32 (28, 38)	

**Weight**, median kg (IQR)	46 (41, 51)	

**BMI**, median kg/m^2 ^(IQR)	16 (15, 19)	

**CD4**^**+ **^**count**, median cells/mm^3^L (IQR)	34 (21, 47)	

**Hemoglobin**, median g/dL (IQR)	9.8 (8.8, 11.6)	

		

**Baseline serum biomarker levels**		

**Phosphate**, median mmol/L (IQR)	1.26 (1.03, 1.42)	[range 0.4-2.3]

**Albumin**, median g/L (IQR)	29.5 (23.9, 33.2)	[range 13.1-49.5]

**Ferritin**, median μg/L (IQR)	221 (59, 485)	[range 4-1332]

**hsCRP**, median mg/L (IQR)	2.8 (1.1, 15.2)	[range 0.2-58.3]

		

**ART regimen, n (%)**		

ZDV/3TC/EFV	8 (6%)	

ZDV/3TC/NVP	34 (24%)	

d4T/3TC/EFV	15 (11%)	

d4T/3TC/NVP	62 (44%)	

TDF/FTC/EFV	4 (3%)	

TDF/FTC/NVP	19 (13%)	

Abbreviations: ART, antiretroviral therapy; BMI, body mass index; hsCRP, highly sensitive C-reactive protein; IQR, interquartile range; 3TC, lamivudine; d4T, stavudine; EFV, efavirenz; FTC, emtricitabine; NVP, nevirapine; TDF; tenofovir; ZDV, zidovudine

Table 2 describes the hazard of mortality or loss to follow up across an observed range of baseline phosphate, albumin, ferritin and hsCRP serum values. A serum phosphate of 1.0 mmol/L was associated with an approximate 20% reduction in the hazard of mortality (adjusted hazard ratio [AHR] 0.79 [95% confidence interval: 0.67, 0.93]) compared with a value of 0.87 mmol/L (i.e., the low end of the normal range) [21]. A serum albumin of 30 g/L compared with 25 g/L was associated with a nearly 50% reduced hazard of mortality (AHR 0.52 [0.38, 0.70]).

**Table 2:** Adjusted hazard ratios for mortality and loss to follow up at 90 days

Baseline serum biomarker	**AHR mortality (95% CI)**^**a**^	p-value	AHR mortality or loss to follow up (95% CI)	p-value
				

Phosphate, mmol/L				

0.87 (ref)	1.00	0.016	1.00	0.855

1.0	0.79 (0.67, 0.93)		0.97 (0.88, 1.08)	

1.5	0.31 (0.14, 0.69)		0.87 (0.53, 1.43)	

				

Albumin, g/L				

25 (ref)	1.00	<0.001	1.00	<0.001

30	0.52 (0.38, 0.70)		0.65 (0.53, 0.79)	

35	0.49 (0.24, 1.01)		0.72 (0.48, 1.08)	

				

Ferritin, μg/L				

25 (ref)	1.00	<0.001	1.00	0.002

250	1.67 (1.30, 2.15)		1.37 (1.15, 1.62)	

1000	9.28 (3.13, 27.50)		3.86 (1.83, 8.17)	

				

hsCRP, mg/L				

5 (ref)	1.00	0.027	1.00	0.103

10	1.56 (1.04, 2.33)		1.27 (1.00, 1.62)	

15	1.96 (1.12, 3.44)		1.41 (1.01, 1.95)	

^a ^Models adjusted for sex and a first principal component (incorporating age, body mass index, CD4^+ ^lymphocyte count, and haemoglobin)

Abbreviation: hsCRP, highly sensitive C-reactive protein

Note: There was evidence that the association between albumin and hsCRP, and the hazard of death was non-linear (p < 0.10), and there was evidence that the association between albumin and the hazard of the combined endpoint of mortality and loss to follow up was non-linear (p < 0.10).

Conversely, higher baseline serum ferritin and hsCRP levels were associated with increased mortality; compared with a ferritin value of 25 μg/L, a baseline serum level of 250 μg/L was associated with an approximate 67% increased hazard of mortality at 12 weeks (AHR 1.67 [1.30, 2.15]), and a value of 1000 μg/L was associated with a nearly 10-fold increased hazard (AHR 9.28 [3.13, 27.50]). A baseline hsCRP level of 15 mg/L was associated with a nearly two-fold increased hazard of death compared with a value of 5 mg/L (AHR 1.96 [1.12, 3.44]).

All adjusted hazard ratios were significantly different from zero (p < 0.05). The calculated hazard of reaching the combined endpoint of mortality or loss to follow up for each gradation of the serum indicators was less pronounced compared with the hazard of mortality alone, and the association was not statistically significant for phosphate or hsCRP.

**Figure 1:**
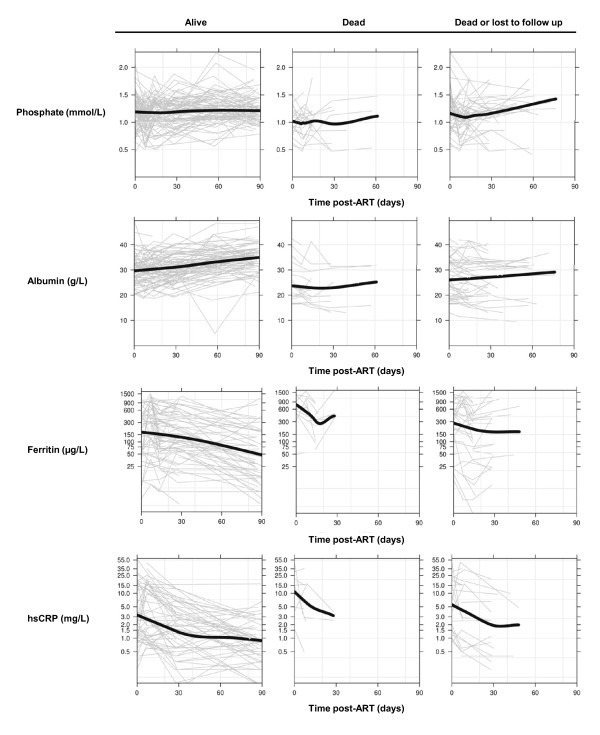
**Biomarker levels among participants alive, deceased, and lost to follow up at 90 days post-ART initiation**. Each grey line represents a single participant; a black line represents the locally weighted scatterplot smoothing (LOWESS) curve.

Figure 1 shows serial phosphate, albumin, ferritin and hsCRP values for each participant alive, deceased or lost to follow up at 12 weeks (grey lines) and the LOWESS curve (black line). Serum phosphate appeared to be relatively stable from ART initiation to 12 weeks among retained survivors, while albumin gradually rose and both ferritin and hsCRP declined. Similar plots among the deceased were truncated, but were notable for lower baseline phosphate and albumin, and higher ferritin and hsCRP levels with steeper initial declines. The baseline values among participants lost to follow up approximated the survivors, but the LOWESS curves showed a drop in phosphate post-ART and no apparent early increase in albumin.

Table 3 describes the association between time-updated serum biomarkers and the attendant hazard of mortality, or the combined endpoint of mortality or loss to follow up, prior to 12 weeks. The Cox model for each biomarker was adjusted for the baseline serum biomarker value, to account for the associations between baseline values and outcome demonstrated in Table 2. The reported adjusted hazard ratios represent the estimated ratio of the hazard of death, at any time prior to 12 weeks, for a hypothetical pair of participants whose serum biomarker values differ by the interval amount (shown in parentheses in the left column).

**Table 3:** Time-dependent predictors of mortality and loss to follow up at 90 days, adjusted for baseline biomarker value

Serum biomarker	Adjusted HR mortality **(95% CI)**^**a**^	p-value	Adjusted HR mortality or loss to follow up (95% CI)	p-value
Phosphate (per 0.1 mmol/L increase)	0.90 (0.77, 1.05)	0.178	0.97 (0.87, 1.07)	0.529

Albumin (per 5 g/L increase)	0.62 (0.43, 0.89)	0.010	0.70 (0.56, 0.87)	0.002

Ferritin (per 100 μg/L increase)	1.05 (0.89, 1.24)	0.536	1.05 (0.93, 1.19)	0.428

hsCRP (per 5 mg/L increase)	1.25 (0.74, 2.12)	0.390	1.33 (0.87, 2.03)	0.184

^a ^Models adjusted for the baseline value of each biomarker, sex, and a first principal component (incorporating age, body mass index, CD4+ lymphocyte count, and hemoglobin).

Abbreviation: hsCRP, highly sensitivite C-reactive protein.

Note: Participants with no lab values were dropped from the Cox models, and time intervals with no corresponding serum level are excluded from analysis.

For interval increases in phosphate, ferritin and hsCRP, the adjusted hazard ratios demonstrated a trend in the same direction as the hazard ratios for interval changes in the baseline biomarker values (Table 2), but the association was not statistically significant for either death or the combined endpoint. However, a 5 g/L increase in albumin was significantly associated with a nearly 40% reduction in the hazard of death (AHR 0.62 [0.43, 0.89]), and an approximate 30% reduction in the hazard of the combined endpoint (AHR 0.70 [0.56, 0.87]).

We previously reported that baseline serum phosphate predicted early mortality in our cohort [13], so we incorporated serum phosphate into the first principal component of the adjusted Cox models of baseline albumin, ferritin and hsCRP to assess for confounding (data not shown). The associations of baseline albumin, ferritin and hsCRP with mortality (shown in Table 2) remained statistically significant (p < 0.01, p < 0.01, and p = 0.03, respectively).

We also repeated the time-dependent Cox analyses by "carrying forward" the last recorded laboratory value for each serum biomarker until the next recorded value or until death, loss to follow up or study termination occurred (rather than dropping intervals without a corresponding value from the analysis, as we have reported here). An interval increase in albumin remained a significant predictor of mortality and the composite endpoint (p = 0.008 and p = 0.001, respectively) while interval changes in phosphate, ferritin and hsCRP remained non-significant.

## Conclusions

We found that pre-treatment nutrition and inflammation serum biomarker levels were associated with mortality prior to 12 weeks among adults with advanced malnutrition and/or immunosuppression initiating ART in a resource-constrained setting. A post-treatment interval change in albumin was also a significant predictor of early mortality, while changes in phosphate, ferritin and hsCRP were not. Our study is the first to describe the relationship of these biomarkers to survival in the immediate post-ART period among patients in the advanced stages of HIV disease, but our findings support similar prior studies investigating longer term health outcomes [7,10-12,16,17,22].

These findings do not imply a causal relationship between elevated inflammation biomarkers or low phosphate or albumin and health outcomes, as individuals with untreated HIV and advanced malnutrition likely have multiple complex metabolic and physiologic derangements. Systemic inflammation in the setting of HIV infection may be a response to occult opportunistic infections, HIV-1 replication, immune reconstitution inflammatory syndrome, or other conditions. Elevated ferritin, in particular, may principally represent a reaction to infectious agents. The pathophysiologic processes contributing to mortality in the immediate post-ART period are a critical research uncertainty and likely differ from those present after several months or years on treatment.

Prior studies have reported greater baseline immune activation in cohorts of HIV-infected and non-infected African adults compared with similar groups in the US and Europe [23-25]. This "background" immune activation may represent opportunistic co-infections (e.g., tuberculosis) or common region-specific infections (e.g., helminthes or malaria parasites), or it could be related to infection with HIV-1 subtype C compared with other viral subtypes [26]. Elevated CRP and other inflammation biomarkers are associated with endothelial dysfunction and an increased risk of cardiovascular events [27,28]; the effect of chronic immune activation on inflammation-related processes (e.g., coronary atherosclerosis) in these populations is an area for further research.

We previously reported that the absolute value of one-week serum phosphate and the change from the pre-treatment level were not significantly associated with mortality in this cohort, as might be observed in the refeeding syndrome [13]. This condition is classically defined by electrolyte and fluid shifts over a period of several weeks in response to increased carbohydrate intake among nutritionally depleted individuals, which predisposes to a range of cardiovascular, respiratory and neurologic sequelae, and death [14,15,29]. The current finding that interval increases in phosphate from treatment initiation to 12 weeks were not associated with reduced subsequent mortality may have been biased toward the null hypothesis by protocol-specified phosphorus supplementation of participants with low levels [13].

Relative long-term (i.e., longer than six month) declines in serum albumin are associated with increased mortality among HIV-infected women (both with and without ART) [16], but absolute albumin values are an uncertain indicator of nutritional status in the presence of chronic inflammation. Albumin is a negative acute-phase reactant, and albumin synthesis, degradation and leakage from the vascular compartment are cytokine-mediated processes [8,30]. Some data suggest systemic inflammation is a stronger determinant of serum albumin levels than either protein intake or body protein stores; however, a lower baseline serum albumin remained significantly associated with mortality at 12 weeks after controlling for baseline hsCRP and ferritin in our cohort (p < 0.01).

The time-dependent analyses of interval changes in phosphate, ferritin and hsCRP demonstrated non-significant hazard ratios that uniformly trended in the same direction as the ratios for the baseline values, suggesting that our study cohort was potentially not large enough to detect a true association. Additionally, our analyses adjusted for potential confounding variables, including sex, age, CD4^+ ^lymphocyte count, BMI and hemoglobin, but residual confounding and/or other unmeasured variables may have affected the associations between serum biomarkers and mortality. Our study investigated relatively few biomarkers of inflammation and nutrition; future studies of early mortality should include a wider range of variables (e.g., pre-albumin, interleukin-6, soluble tumor necrosis factor receptors and ligands, monocyte chemoattractant protein-1, urinary F-2 isoprostane, and markers of T cell activation, among others).

Our cohort also had a high rate of participant attrition, but the observed loss rates are similar to reports from other programmatic cohorts in sub-Saharan Africa [31]. It is likely that many of the lost participants represented unrecorded deaths or those who ceased treatment and subsequently died. The presence or absence of occult opportunistic infections could not be adequately explored in this study because of limited diagnostic capabilities available in public-sector clinics in Lusaka, Zambia. Finally, future studies would benefit from careful ascertainment of a cause of death for each participant, principally to determine the prevalence of occult infections or inflammation-associated, non-AIDS-defining events.

In this observational cohort study, pre-treatment phosphate, albumin, ferritin and hsCRP serum levels were associated with mortality in the first 12 weeks on ART, but only the interval change in post-treatment albumin was associated with this outcome, while changes in the remaining biomarkers were not. The presence of increased baseline inflammation biomarkers among those deceased at 12 weeks may indicate the presence of undiagnosed secondary infections, such as tuberculosis, fungi and parasites.

Measurement of pre-treatment phosphate, albumin, ferritin and hsCRP serum levels may have a role in the risk stratification of low BMI adults starting ART, and in the future, could prompt additional diagnostic procedures, trials of anti-inflammatory therapies, nutritional supplementation, or other targeted intervention strategies. The role of post-treatment monitoring of phosphate and inflammation biomarkers should be explored in larger studies.

Low BMI individuals represent a considerable proportion of adults requiring HIV care in resource-constrained settings, and further study of the metabolic and physiologic derangements, co-morbid infections, and potential therapeutic interventions to improve outcomes in this vulnerable population are necessary.

## Competing interests

The authors declare that they have no competing interests.

## Authors' contributions

DH, CN, EK, IZ and LM were responsible for study design and data collection. MB, BS and JK performed the statistical analyses. JC and EK performed the laboratory analyses. JK, MB, EK, CW, JC and AM drafted the manuscript, which all authors reviewed, edited and approved.
